# The association between *rs12885713* polymorphism in *CALM1* and risk of osteoarthritis: A meta-analysis of case–control studies

**DOI:** 10.1097/MD.0000000000012235

**Published:** 2018-09-07

**Authors:** Jia Shi, Shu-tao Gao, Zheng-tao Lv, Wei-bin Sheng, Hao Kang

**Affiliations:** a Department of Orthopedics, Tongji Hospital, Tongji Medical College, Huazhong University of Science and Technology, Wuhan, Hubei; b Department of Spine Surgery, The First Affiliate Hospital of Xinjiang Medical University, Urumqi, Xinjiang, China.

**Keywords:** *CALM1*, meta-analysis, osteoarthritis, polymorphism, *rs12885713*, systematic review

## Abstract

**Objective:**

The single nucleotide polymorphism (SNP) *rs12885713* of calmodulin 1 gene (*CALM1*) has been reported to involve in the etiology of osteoarthritis (OA) in several association studies with limited sample size and conflicting results. The purpose of the present systematic review and meta-analysis was to evaluate and synthesize the currently available data on the correlation between *rs12885713* and OA susceptibility.

**Methods:**

Six electronic databases including PubMed, EMBASE, ISI Web of Science, CNETRAL, CNKI, and Wanfang were systematically retrieved to identify relevant observational articles published before October 2017. Summary odds ratios (ORs) and corresponding 95% confidence intervals (95% CIs) were calculated to indicate the association between *CALM1* polymorphism and OA. Risk of bias was assessed through the Newcastle-Ottawa Scale. Predetermined subgroups and sensitivity analyses were performed using the RevMan 5.3 software. Publication bias was evaluated by Egger and Begg tests.

**Results:**

Overall, 5 case–control studies involving 2183 OA patients 2654 healthy control subjects satisfied the meta-analysis. Recessive model was confirmed to be the best-matching genetic model (TT + TC versus CC). The pooled outcomes indicated that *rs12885713* SNP was not significantly associated with OA vulnerability (OR 1.11, 95% CI 0.97, 1.27; *P* = .12). When stratified by different genders, OA sites, and population descents respectively, still non-significant associations were found.

**Conclusion:**

Based on the findings of our present study, the *rs12885713* polymorphism of *CALM1* did not appear to be associated with OA predisposition.

## 1 Introduction

Osteoarthritis (OA), mainly characterized by gradual damage of articular cartilage, synovial membrane, subchondral bone, ligament, joint capsule, and even the peri-articular muscles and tendons, is the most common type of arthritis and a leading cause of disability in elderly adults worldwide.^[1]^ OA can arise in various synovial joints, but mostly affects the weight-bearing joints like knees, hips, and spine.^[2]^ It is reported that approximately 10% men and 13% women aged 60 and over suffer symptomatic knee OA.^[3]^ The incidence and prevalence of OA increase steeply with age, especially with the prolongation of human being lifespan over recent years.^[4]^ OA is increasingly becoming a serious public-health problem.^[5]^ Current epidemiological situation of OA has a noteworthy impact on society.^[6]^

The precise etiopathogenesis underlying OA remains unclear. Risk factors for OA include age, gender, abnormal loading, obesity as well as genetics.^[5]^ Among them, genetic factors might play a pivotal role and have received high attention.^[7]^ A latest large-scale twin study across the UK has revealed a significant contribution of genetic factors with an estimated heritability of 37% to 65%.^[8]^ Other reports from Finland and America also highlighted the importance of OA heritability.^[9,10]^ Taken together, these studies indicated the heritability of OA can be no less than 50%, suggesting that half the mutation in vulnerability to OA among population attribute to genetic factors.^[7]^ During the past 2 decades, considerable candidate genes with innumerous gene polymorphisms have been reported as concealing risk alleles for OA vulnerability. The candidates have been selected predominantly according to the principle that the product they code for is regulators of both joint formation and maintenance.^[11]^ A great many of polymorphisms in various genes such as *ESR1*,^[12,13]^*VDR*,^[14,15]^*ADAM12*,^[16,17]^*MMP3*,^[18]^ and *Asporin*^[19]^ have been demonstrated to be related with risk of OA.

Calmodulin (CaM), a widely distributed Ca^2+^ receptor protein, regulates a range of signaling processes like cell proliferation and differentiation, cytoskeleton architecture and function, and metabolic homeostasis in eukaryotic cells. It is well-known that mechanical compression of articular chondrocytes can cause changes in *Aggrecan* gene expression, and relevant changes mainly rely on CaM signaling.^[20,21]^ CaM is coded by 3 distinct CaM genes (*CALMs*), namely *CALM1*, *CALM2*, and *CALM3*.^[22]^*CALM2* has been reported to be associated with hip OA in Japanese population.^[23]^ Several studies also demonstrated the relationship between *CALM1* and OA vulnerability with conflicting result.

Most genes contributing to OA confer simply a very modest increase or decrease in the risk, and most of the gene-disease association studies used sample-sizes of hundreds but not thousands of cases and controls, they consequently lack the power to detect such associations. Compared to individual study, a meta-analysis can be conducted to enhance the statistical power of the association by enlarging the sample size and acquire a more accurate estimation. Therefore, we performed this study to investigate the association between *CALM1* polymorphism and risk of OA using a meta-analysis approach.

## 2 Methods

This systematic review and meta-analysis were conducted following the Preferred Reporting Items for Systematic Reviews and Meta-Analyses (PRISMA) guidelines.^[24]^ This systematic-review and meta-analysis do not need ethical approval because it does not include data linked to individual patient information.

### 2.1 Literature search strategy

The literature search of this study was conducted on 6 online electronic databases including PubMed, EMBASE, ISI Web of Science, CNETRAL, CNKI, and Wanfang, with all articles published previous to October 2017. A combination of Medical Subject Headings (MeSH) together with free terms and was used to retrieve all the potentially eligible publications. No language restrictions were imposed. The following search strategy was used in English databases: (Single Nucleotide Polymorphism [SNP] or polymorphism or SNPs or “Polymorphism, Single Nucleotide”[MeSH]) and (*calmodulin* or *CALM1*) and (OA or osteoarthrosis or osteoarthritides or “Osteoarthritis”[MeSH]). For Chinese academic databanks, we used “duo tai xing” together with “*CALM1*” to identify related Chinese articles. The bibliographic lists of relevant reviews and full-text articles were also manually searched to capture additional possible studies.

### 2.2 Inclusion and exclusion criteria

Studies meeting the following criteria were included for review:

(1) studies that assessed the correlation between the *rs12885713* polymorphism in *CALM1* and OA susceptibility that have been published;
(2) studies should be designed based on a case-control design;
(3) the diagnosis of OA should be made according to well-established diagnostic criteria and confirmed by radiographic images, subjects in control groups should be without any history or sign of OA;
(4) studies were required to provide sufficient data to calculate the odds ratio (OR) with its 95% confidence interval (95% CI).

Correspondingly, studies satisfying the following criteria were excluded:

(1) animal studies, review, expert opinion, conference abstract, case report, or case series;
(2) data that overlapped with previous publications;
(3) studies failed to provide sufficient data to evaluate OR and the associated 95% CI.

### 2.3 Methodological quality assessment

The methodological quality of eligible studies was assessed independently by 2 reviewers (J. Shi and S. Gao) using the Newcastle-Ottawa Scale (NOS) for observational studies.^[25]^ Three broad perspectives including selection of cases and controls, comparability of the groups and ascertainment of outcome of interest were evaluated using the Star system. Discrepancies between reviewers were settled through discussion until a mutual consensus was reached.

### 2.4 Data extraction

Two investigators (J. Shi and S. Gao) conducted the reading and data extraction of all the included studies independently. A standardized data extraction list was utilized to collect information including first author, year of the publication, country, ethnicity of enrolled subjects, diagnosis of cases, genotypes distribution of cases and controls, Hardy–Weinberg Equilibrium (HWE) for control subjects in each study. In the event of any discrepancy through the process, 2 reviewers reinvestigated the article together and discussed with each other until coming to an agreement.

### 2.5 Statistical analysis

Departure from HWE was evaluated by using Chi-square test to assess goodness of fit in control subjects of each included study. The strength of association between *rs12885713* polymorphism in *CALM1* and risk of OA was indicated as OR along with the associated 95% CI. In order to avoid an inflated Type I error rate, we did not perform any assumptions about the genetic model of inherence in advance. The most appropriate genetic model for *CALM1* polymorphism in the risk of OA was determined by a model-free approach.^[26]^ Briefly, OR1, OR2, and OR3 were calculated for genotypes TT versus CC, TC versus CC, and TT versus TC to capture the magnitude of genetic effect. Then the most plausible genetic model of inherence was determined according to the relationships between the 3 pairwise comparisons as follow:

(1) Recessive model: if OR1 = OR3≠1 and OR2 = 1;
(2) Dominant model: if OR1 = OR2≠1 and OR3 = 1;
(3) Complete over-dominant model: if OR1 = 1, OR2 = 1/OR3≠1;
(4) Codominant model: if OR1 > OR2 > 1 and OR1 > OR3 > 1, or OR1 < OR2 < 1 and OR1 < OR3 < 1.

After that the underlying genetic model was confirmed, the counts of genotypes were collapsed into 2 categories to obtain the merged results. The between-study heterogeneity was assessed using the Q-statistical test and I^2^ test.^[27]^ The random-effect model and fixed-effect model were used for data combination in the presence (*P* < .1, I^2^ > 50%) or absence of heterogeneity (*P* > .1, I^2^ < 50% indicates acceptable heterogeneity) respectively. In case of statistically significant heterogeneity across studies, subgroup-analysis by ethnicity, diagnosis, and gender were performed to find the possible source of heterogeneity.^[28,29]^ The leave-one-out sensitivity analysis was conducted by removing each study in turn and reassessing the resulting effect on the overall effect. Power analysis was performed using the Power and Sample Size Calculation (PS) program (http://biostat.mc.vanderbilt.edu/wiki/Main/PowerSampleSize) to evaluate whether our meta-analysis could offer adequate power to detect the association between *CALM1* polymorphism and risk of OA at a level of significance of 0.05. Egger regression test and Begg rank correlation test were used to estimate the publication bias (Stata version 12.0, Stata Corp LP).^[30]^ Forest plots and funnel plots were generated using RevMan 5.3 software (Copenhagen: The Nordic Cochrane Centre, The Cochrane Collaboration, 2014).

## 3 Results

### 3.1 Literature search

**Figure 1:**
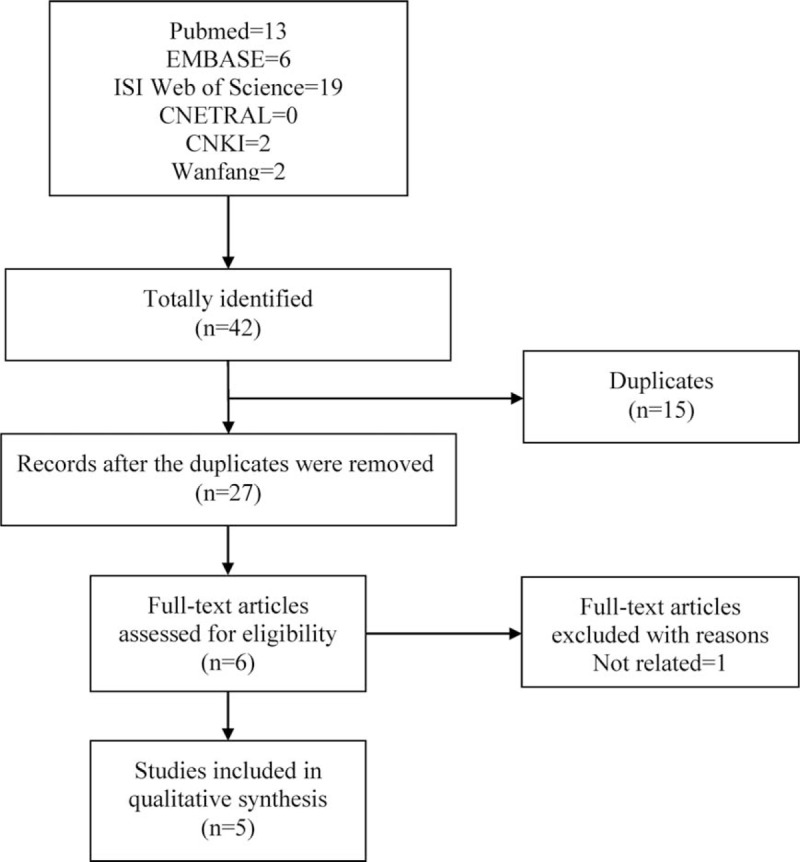
Flow diagram of literature search and screen.

Six online databases were searched for potentially relevant studies and the initial literature search yielded a total of 42 records including 13 from PubMed, 6 from EMBASE, 19 from ISI Web of Science, 2 from Wanfang and 2 from CNKI. After the integration of these records, 15 duplicated records were removed and the remaining 27 were screened for titles and abstracts for eligibility. According to the predetermined inclusion and exclusion criteria, 21 irrelevant studies were excluded and the remaining 6 studies were subject to a full-text screen. Finally, 1 study ^[23]^ was removed for irrelevance and 5 studies^[31–35]^ were deemed eligible and included for meta-analysis. The process of literature search was presented in Figure 1.

### 3.2 Main characteristics

The main characteristics of included studies were presented in Table 1. Five case-control studies involving a total of 2183 OA patients 2654 healthy control subjects were identified and included in our meta-analysis. The studies were performed in the UK,^[34]^ India,^[33]^ Greece,^[32]^ China, ^[31]^ and Japan,^[35]^ respectively, the sample-size ranged from 351 to 1672. Three of 5 included studies^[31–33]^ focused on the association between *rs12885713* and KOA while the remaining 2 studies^[34,35]^ featured to assess the association in HOA. The clinical stage of OA was assessed by radiographic images according to the Kellgren–Lawrence classification system. The genotype frequencies of case and control groups were summarized in Table 1, the control subjects of each included study were in HWE.

**Table 1:**
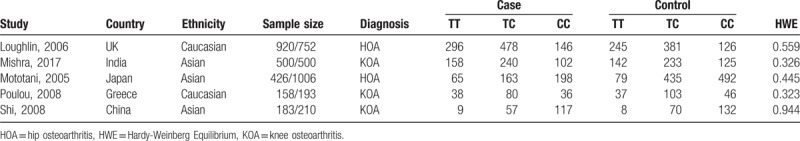
Main characteristics of included studies and genotype frequencies in case and control groups.

According to the NOS, our included studies achieved an average of 5.6 stars for methodological quality assessment (Table 2).

**Table 2:**
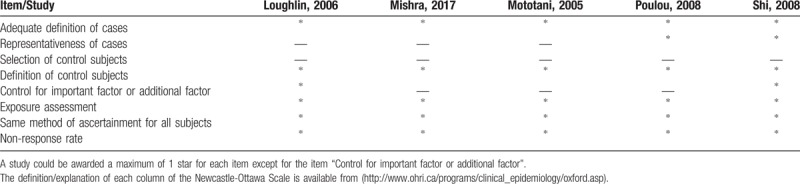
Quality assessment of included studies according to the Newcastle-Ottawa Scale.

### 3.3 Meta-analyses and subgroup-analyses

**Figure 2:**
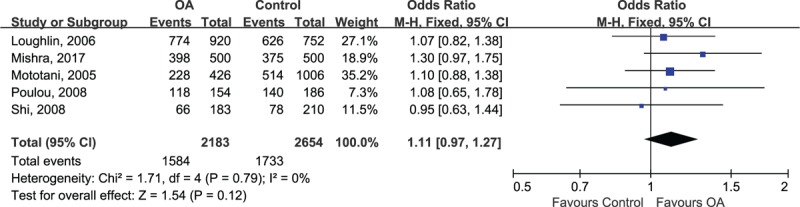
Forest plot of *rs12885713* polymorphism and risk of OA. OA = osteoarthritis.

Before pooling data from each individual study, we made testable hypotheses about the best-matching genetic model of inherence. The estimated OR1 (TT/CC: 1.34, 95% CI 1.12, 1.61; *P* = .001) and OR3 (TT/TC: 1.16, 95% CI 1.00, 1.35; *P* = .047) were both statistically significant whereas the estimated OR2 (TC/CC: 1.03, 95% CI 0.90, 1.19; *P* = .64) was insignificant, suggesting that the recessive model was the most plausible genetic model for meta-analysis. When using the recessive model, the counts of genotypes of TT and TC groups were combined and compared with CC groups. Since there was no heterogeneity among included studies (*P* = .79, I^2^ = 0%), the fixed-effect model was used for statistical analysis. The combined ORs showed that there was no statistically significant association between *rs12885713* in *CALM1* and risk of OA (OR 1.11, 95% CI 0.97, 1.27; *P* = .12) (Fig. 2).

Although there was no heterogeneity across included studies, we performed subgroup-analysis by ethnicity (Asian and Caucasian), gender (female and male) and diagnosis (HOA and KOA) to evaluate the association between *rs12885713* and risk of OA in different subgroups (Table 3). However, there were no statistically significant associations of *CALM1* polymorphism and OA in any ethnicity groups or gender, the association remained insignificant regardless of the sites of OA.

**Table 3:**
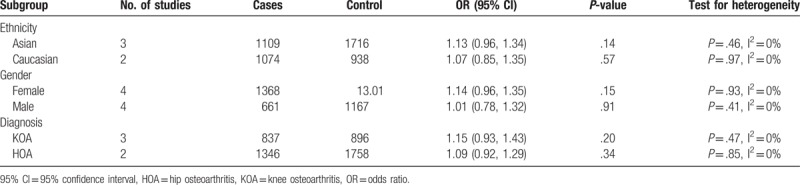
Subgroup-analyses results.

### 3.4 Sensitivity analysis and publication bias

**Figure 3:**
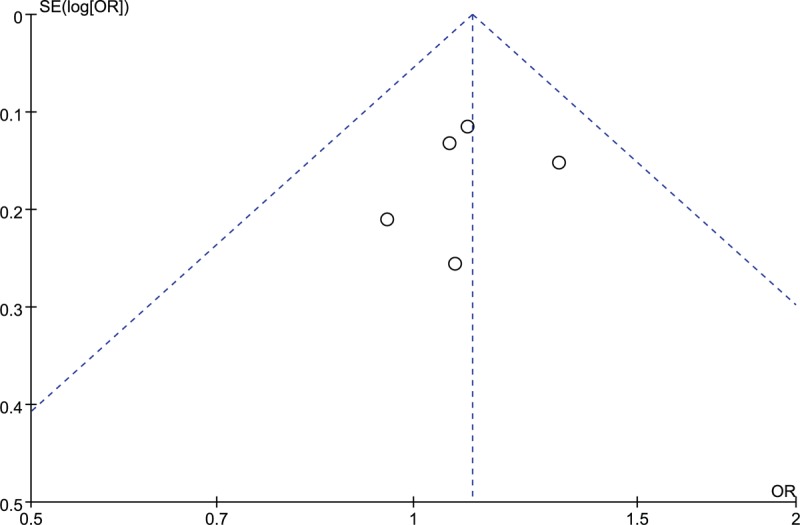
Funnel plot of *rs12885713* polymorphism and risk of OA. OA = osteoarthritis.

The leave-one-out sensitivity analysis confirmed the robustness and reliability of our drawn conclusion. The association between *rs12885713* polymorphism and risk of OA remained insignificant after the removal of any included study (detailed data not shown). The funnel plot was symmetrical (Fig. 3), the Egger test (t = -0.37, *P* = .735) and Begg test (z = 0.24, *P* = .806) also suggested no statistically significant publication bias. The result of power analysis suggested that our meta-analysis had a power of 1.00 to detect the association between *CALM1* polymorphism and risk of OA at a level of significance of 0.05.

## 4 Discussion

Despite the etiopathogenesis of OA is multifactorial and largely undetermined, both intrinsic factors and extrinsic factors are considered to be etiology. To some extent, epidemiological studies have encouraged us to quantify the contribution of those individually independent risk factors to OA. Owing to the predominant effect of genetic factors on OA, a better understanding of gene polymorphisms correlated to OA liability enable us to predict the disease and take preventive measures, as well as identify potential targets for specific pharmacologic treatment. Based on the findings of our present study, *rs12885713* polymorphism in *CALM1* was not statistically associated with risk of OA, regardless of the OA diagnosis, gender or ethnicity.

As an intrinsic genetic factor, the *CALM1* has been reported to contribute greatly to hip OA both *in vivo* and *ex vivo* studies. *CALM1* is located at human chromosome 14q32.11. This gene along with *CALM2* and *CALM3* is responsible for the encoding of CaM, a type of highly conserved and ubiquitous Ca^2+^ binding protein controlling Ca^2+^-dependent processes in higher vertebrates.^[36]^ Valhmu and coworkers^[21]^ found that CaM involved in the metabolic activity in the bovine articular chondrocytes under mechanical stimulation. Mototani et al^[35]^ observed that CaM implicated in chondrogenesis through regulating the expression of *COL2A1* and *AGC1* genes whose expression products are type II collagen and aggrecan, both of which are principal component of extracellular matrix in cartilage. In addition, they also demonstrated the *CALM1* expression level in human OA cartilage tissue was significantly higher than that in cultured normal human articular chondrocytes.^[35]^ Mishra et al^[33]^ reported that the reduced mRNA and protein expression level of *CALM1* in the peripheral blood lymphocytes from OA patients appeared to be parallel to the decreased formation of cartilage as well as its disruption. These findings altogether suggested that *CALM1* participated and played an important role in the OA pathogenesis.

Albeit a negative relationship in our study, the association between *rs12885713* polymorphism and other gene variants cannot be denied. Under most circumstance, a gene functions through an intricate web of mechanisms, this rule also applies to gene polymorphisms. Just as Mishra et al have observed the distribution frequencies of the TGC haplotype that carries 3 variant alleles of *T-rs12885713* and *G-rs3814843* and *C-rs2300496* among OA patients were significantly higher compared to the normal controls. Besides, when combining different polymorphic loci from *ESR-a*, *CALM1,* and *GDF5*, the variant genotype frequencies in OA patients were as well higher than the controls. What is more, Mototani et al and Poulou et al observed the combinatorial interplay between 2 loci from *CALM1* and *ASPN*.^[32,35]^

The most robust evidence for a veritable association between a specific gene and a certain disease ought to derive from well-designed studies with large sample. Only such evidence is able to withstand the confirmation of later replication studies. By a large-scale association study accompanied with the subsequent functional investigations, Mototani et al^[35]^ initially reported the association between *rs12885713* polymorphism of *CALM1* core promoter and hip OA risk, another 4 comparable studies were also conducted among different ethnicities and population. However, subsequent replication studies performed in Caucasian, Chinese, and Indian populations failed to replicate positive results. These inconsistent results further emphasized the diversity and complexity of genetic impact on OA. To increase the statistical power, we collected the currently available evidence and conducted a comprehensive synthesis of these data. In the summarized analysis of 2183 OA patients 2654 healthy control subjects, our main findings indicated the *rs12885713* polymorphism did not appear to be associated with OA risk under the best-matching recessive model. Considering genetic factors have been reported to function differently between the 2 genders,^[9]^ at distinct sites of the body,^[8]^ and among diverse population, we conducted subgroup analysis according to the corresponding differences. Nevertheless, there was still non-significant association could be detected, indicating that the *rs12885713* polymorphism is not a likely liability locus for OA when examined in isolation.

For the most part, most genes contributing to a certain disease confer only a very modest increase or decrease in risk of disease. Thus, to detect these gene-disease associations with adequate statistical power requires large sample size. To avoid yielding an inflated Type I error by using multiple genetic models of inheritance, we adopted a model-free approach without assuming a genetic model in advance. The association between *CALM1* polymorphism and risk of OA remained insignificant under the most plausible genetic model, the results were strengthened by no statistical between-study heterogeneity (I^2^ = 0%) and validated by the leave-one-out sensitivity analysis. The initial study of association between *CALM1* and risk of OA may have been a false-positive result due to chance or systemic bias existed in the corresponding study. According to the methodological quality assessed using NOS, Mototanis’ study achieved 5 stars, while the investigators failed to report the controlling for important factors, the representativeness of cases in the study was also insufficient.^[35]^ Additional association studies and further functional studies are warranted to confirm the real association between *CALM1* polymorphism and risk of OA.

Several limitations in this study should not be neglected when interpreting the results. First, all included studies used sample of hundreds but not thousands of cases and controls, they may lack the ability to detect the association between *CALM1* polymorphism and risk of OA, especially when the overall effect is marginal. Second, only 5 studies were included in our meta-analysis, the number of included studies was relatively small. Third, 4 of the 5 included studies were hospital-based, thus it was difficult to avoid the overestimate of OR caused by high selection of participants. Fourth, 2 factors were considered to contribute to the null association of our present study. On the 1 hand, perhaps the comparatively limited sample size of our study was not powerful enough to obtain a significant association; on the other hand, of these included studies, participants did not share the same ethnic origin, age was variable, OA site was different as well. Such clinical heterogeneity might also conceal the actual association. If *rs12885713* contributes predominantly to a specific subtype of OA such as KOA and HOA, then the pooled results from different studies will possibly affect the power of each study to detect the real association. Population heterogeneity across included studies could result in differences in study outcomes as well. In the subgroup-analysis by ethnicity, Asian included Chinese, Japanese and Indian. Although a previous meta-analysis^[37]^ of replicated genetic association studies reported that even when the SNP frequency differs across populations, the overall effect of mutations remains approximately constant, the negative findings of our current meta-analysis should be interpreted with caution. However, although some limitations existed in our present study, the overall methodological quality of included studies was moderate and the data from each individual study were consistent. A reasonable degree of confidence should be given to the null association between *CALM1* polymorphism and risk of OA based upon the results of our meta-analysis. Additional association studies with larger sample size within other ethnicity backgrounds are strongly encouraged.

## Author contributions

Hao Kang produced the idea to this study and he was responsible for making the final version of this paper. Jia Shi, Shu-tao Gao, and Zheng-tao Lv did the literature search, screened the potentially eligible studies and evaluated the data from each included study, they contributed equally to this work. Wei-bin Sheng critically revised this manuscript, including important intellectual contents and the grammatical mistakes existed in our original study. Hao Kang had full access to all of the data in the study and takes responsibility for the integrity of the data and the accuracy of the data analysis.

**Conceptualization:** Hao Kang.

**Data curation:** Jia Shi, Shu-tao Gao, Zheng-tao Lv, Hao Kang.

**Formal analysis:** Jia Shi, Shu-tao Gao, Zheng-tao Lv.

**Funding acquisition:** Hao Kang.

**Investigation:** Jia Shi, Shu-tao Gao, Zheng-tao Lv.

**Methodology:** Jia Shi, Shu-tao Gao, Zheng-tao Lv.

**Project administration:** Zheng-tao Lv.

**Resources:** Jia Shi, Shu-tao Gao, Zheng-tao Lv.

**Software:** Jia Shi, Shu-tao Gao, Zheng-tao Lv.

**Supervision:** Zheng-tao Lv, Wei-bin Sheng, Hao Kang.

**Validation:** Jia Shi, Zheng-tao Lv, Wei-bin Sheng, Hao Kang.

**Visualization:** Jia Shi, Shu-tao Gao, Zheng-tao Lv.

**Writing – original draft:** Jia Shi, Shu-tao Gao, Zheng-tao Lv, Wei-bin Sheng, Hao Kang.

**Writing – review & editing:** Jia Shi, Shu-tao Gao, Zheng-tao Lv, Wei-bin Sheng, Hao Kang.

Zheng-tao Lv: 0000-0002-8238-7194.
